# Poison-Oak Eruption

**Published:** 1876-09

**Authors:** E. H. Bernard


Poison-Oak Eruption—*By E. H. Bernard.*—I have just read in your journal an article by Professor L. P. Yandell, Jr., on "Poison Oak." I think I can still add one to the list of reliable remedies for the torment inflicted by that infernal thing; to-wit, fluid extract of gelsem. sempervirens, applied by simply brushing it over the affected part with a feather.

Some few weeks ago I had to prescribe for a boy who had got the poison, of all places in the world, all over the genital organs. The burning sensation invariably came on about sunset, and tormented the patient all night long, subsiding always at daylight. (Here was a good hint to try quinine, but I failed to use it.) After trying about a dozen of so-styled "infallibles," I heard of the gelseminum, and concluded to try it; for I was just then "at the end of my row," and badly bothered to find a remedy. The first application gave complete relief, and the patient slept soundly all night. After that the gelseminum was applied three more times, and that ended the job; and a most devilish, troublesome job it was until the gelsiminum let me out of the scrape.

Some thirty years ago I saw sulphate of copper (two grains to one ounce of water) used in a great number of cases, and with general success; but lately I have known it to fail several times. Should I ever be called upon to treat another case, I would certainly try quinine at the start. The gelseminum treatment may be already known to many or all readers of the *News;* but if not, they are abundantly welcome to the hint. I do not propose to take out a patent for it just at present.—*Louisville Medical News.*

